# Effective scale-up: avoiding the same old traps

**DOI:** 10.1186/1478-4491-7-2

**Published:** 2009-01-14

**Authors:** Pape A Gaye, David Nelson

**Affiliations:** 1 IntraHealth International, Inc., 6340 Quadrangle Drive, Suite 200, Chapel Hill, NC 27517, USA

## Abstract

Despite progress in developing more effective training methodologies, training initiatives for health workers continue to experience common pitfalls that have beset the overall success and cost-effectiveness of these programs for decades. These include lack of country-level coordination of health training, inequitable access to training, interrupted services, and failure to reinforce skills and knowledge training by addressing other performance factors. These pitfalls are now seen as aggravating the current crisis in human resources for health and impeding the effective scale-up of training and the potential impact of promising strategies such as task shifting to address health worker shortages. Drawing on IntraHealth International's lessons learned in designing reproductive health and HIV/AIDS training and performance improvement programmes, this commentary discusses promising practices for strengthening human resources for health through more efficient and effective training and learning programmes that avoid the same old traps. These promising practices include the following:

Assessing performance gaps and opportunities before designing a training initiative; addressing performance factors other than skills and knowledge that health workers need to perform well; applying a "learning for performance" approach; standardizing curricula throughout a country; linking pre-service education, in-service training and professional associations; enhancing traditional education; strengthening human resources information systems to improve workforce planning, policies and management; applying technology to meet training needs.

## Background

Despite the evolution of training approaches and technology, training initiatives for health workers continue to experience the same pitfalls, all at great cost and contributing to inadequate production and retention of the needed health workforce. Since the 1970s, training designs have progressed from information-based, classroom-oriented models to more interactive, competence-based approaches to performance-based training methodologies that emphasize effective transfer of skills and knowledge to the workplace. The trend toward more holistic and supportive training programmes has generally produced stronger on-the-job results among trained health workers. Still, training initiatives often fall into the same old traps that have beset the overall success and cost-effectiveness of these programmes for decades.

With the current crisis in human resources for health (HRH), these pitfalls have become more serious and are now seen as aggravating the situation and impeding the effective scale-up of training. On 9 January 2008, participants in a meeting with the World Health Organization (WHO), ministers of health, development partners, nongovernmental organizations (NGOs) and people living with HIV/AIDS embraced the Addis Ababa declaration, a call to action for the adoption of new WHO guidelines and recommendations on task-shifting as one of the strategies for bringing solutions to the HRH crisis. As described in the guidelines, task-shifting involves redistributing tasks, as appropriate, "from highly qualified health workers to health workers with shorter training and fewer qualifications in order to make more efficient use of the available human resources for health." [1]. Successful implementation of these guidelines will require addressing the common pitfalls to training initiatives. Among the major traps are the following:

• *Lack of country-level coordination of health training among donors, partners, ministries and other key actors*: This manifests itself in many ways, among them mismatches between the skills and knowledge required by a country's health systems and the skills and knowledge produced by its educational systems. At its extreme, poor coordination and management of training can result in providers' spending more time in training than offering the services they are trained to deliver.

• *Inequitable access to training*: for reasons such as gender, type of cadre and location of the health worker.

• *Interrupted services*: The tendency to bring health workers to centralized locations for training too often causes serious disruptions in service delivery at facilities serving the most vulnerable populations.

• *Failure to reinforce skills and knowledge training by addressing other performance factors*: These factors include the work environment (equipment, supplies and other tools needed to provide services of good quality), organizational support, clear expectations and feedback, and motivation. Lack of attention to these factors hampers the effectiveness of training programmes, leads to poor application of newly-acquired learning in the workplace and can discourage retention of trained workers.

This commentary presents some key factors to consider for effective and accelerated scale-up of holistic training and performance-improvement programmes, drawing on IntraHealth International's lessons learned in designing reproductive health and HIV/AIDS training and performance improvement programmes over the last 28 years in countries around the world. Our work in human resources for health, especially through leading the USAID-funded Capacity Project, also informs this article.

## Discussion

Based on our experience, promising practices for strengthening HRH through more efficient and effective training and education programmes include the following.

### Assessing performance gaps and opportunities

IntraHealth's experience conducting health worker performance needs assessments in more than 20 countries has found such assessments invaluable in identifying the skills and knowledge gaps to address and the opportunities to exploit in training and education initiatives and in determining which categories of workers are needed to meet priority health care needs. Assessments reveal such essential information as the variety of skill levels that are needed at a point of service delivery, and the factors other than skills and knowledge that must be addressed to improve health worker performance and service quality. Performance needs assessments can often be accomplished in a short time and without great expense [2].

### Addressing all performance factors

Even the best training and education programmes will prove ineffective if factors other than skills and knowledge that health workers need to perform their jobs well are not consistently in place. These factors–drawing on decades of private-sector experience with quality improvement and refined and promoted among USAID-funded agencies by the Performance Improvement Consultative Group–include adequate equipment, workspace and commodities; clear job descriptions and expectations; motivation and incentives to perform as expected; supportive supervision; and clear and immediate performance feedback [3]. In the context of task-shifting, it is also important to focus on the policy and regulatory environment that facilitates the use of community-based and other nontraditional providers. Combinations of training and non-training approaches are most effective when they are based on performance behaviors, learning needs and systematic instructional design as well as contextual and cultural factors that can affect workplace performance.

### Learning for performance

Training curricula are frequently burdened with too much content, diluting learning related to job performance [4]. When learning interventions are relevant to specific job responsibilities and tasks, health workers may be more engaged and involved in learning and more motivated to perform well on the job. By focusing on essential content, skills and knowledge while delivering specific outcomes, the "learning for performance" approach [5] is especially well suited to the education and training efforts required to support key HRH strengthening processes, including reaching more nontraditional providers. These processes include aligning training with national health goals and priorities, accelerating the training and rapid deployment of health workers, creating and deploying new health worker categories, shifting or redistributing tasks among existing categories or to new categories, and developing fast-track bridging programmes to advance health workers to positions in higher-priority categories.

### Standardizing curricula

Especially as curricula are simplified and focused to address urgent HRH goals and service priorities, it is essential that they are linked and aligned with national training standards, protocols and policies, and are standardized and replicable throughout a country to promote quality and consistency in outcomes.

### Linking pre-service education, in-service training and professional associations

While sometimes slow or difficult to bring about, these linkages can reduce redundancies, help balance training needs and lessen the burden of multiple, vertical training programmes on the workforce. In addition, strengthening the role of professional associations can help promote high standards of practice, advocate policy change and empower female-dominated health professions [6].

### Enhancing traditional education

Taking advantage of opportunities to develop health workers' professional skills, behaviours and attitudes–both during and outside of training and education programmes–can enhance learning and promote retention and improved service delivery [7]. Examples of areas for professional development include business and management skills, peer group support networks, lifelong learning skills and sensitivity to gender issues.

### Strengthening human resources information systems

Are a country's health workers employed in facilities that match their education and training? Are health workers optimally deployed in locations to meet national health priorities? Are they receiving appropriate in-service training? A strong human resources information system provides the data health care leaders and managers need to answer key policy questions affecting health care service delivery and to plan rationally for who should be trained and in what areas. An exciting component of the Capacity Project is the development of free, Open Source software applications [8] that countries can use to track health worker training, certification and licensure; maintain personnel information; model long-term health workforce needs; and inform policy analysis and development related to such factors as recruitment, deployment and retention. These software applications can and should also be used to track and provide data on community and other non-facility-based providers for more effective workforce planning and support. The Open Source solutions offer great promise for decreasing implementation and ongoing maintenance of these systems, as well as providing a global community for support and continuous improvement.

### Applying technology to meet training needs

When the right technology is matched to the situation, it can be applied effectively for learning in low-resource settings. A variety of approaches–from cell phones and personal digital assistants (PDAs) to digital video discs (DVDs) and e-learning courses combining self-study and peer review and support–should be taken into consideration in planning training initiatives that can address such issues as minimizing impact on service delivery and expanding access to remote areas.

Two recent publications provide useful frameworks of steps and phases for successful scale-up [9,10]. Regardless of which framework is used, additional emphasis should be placed on the following key factors in accelerating the process of scale-up:

### Identifying and nurturing champions

The training-of-trainers approach has proven to be an effective means of spreading new information and best practices. This approach can be used provided the formation of teams of trainers includes representatives from both the in-service and pre-service sectors as well as from service delivery.

### Engaging stakeholders

The promotion of training beyond the classroom often meets resistance from decision-makers and health care providers who view such centralized training events away from their work sites as opportunities for motivation. Truly engaging these stakeholders in the dialogue about more effective approaches is a good way to address this issue and to foster local ownership of the scale-up process.

### Ensuring coordination of training activities

Stakeholder engagement is an important first step towards addressing the coordination challenges noted earlier as a common pitfall. However, successful coordination of training also requires continued monitoring and oversight at the local level.

## Conclusion

With the global shortage of health workers undermining health care delivery systems in many countries, the critical effort to scale up training and education for health workers demands vision, knowledge sharing and tools to avoid common pitfalls and to consider each training opportunity in the broader context of strengthening human resources for health. Based on one organization's experience, the key factors discussed in this commentary offer promising practices that can facilitate more effective, rapid and efficient training initiatives that avoid the same old traps.

## Competing interests

The authors declare that they have no competing interests.

## Authors' contributions

DN drafted and revised the manuscript. PG made substantial contributions to content conceptualization and development and revisions.

## References

[B1] World Health Organization (2008). Task Shifting: Rational Redistribution of Tasks among Health Workforce Teams: Global Recommendations and Guidelines Geneva.

[B2] PRIME II Project (2004). Improving the Performance of Primary Providers in Family Planning and Reproductive Health Results and Lessons Learned from the PRIME II Project, 1999–2004.

[B3] Performance Improvement Stages, Steps and Tools. http://www.intrahealth.org/sst.

[B4] Task Force for Scaling Up Education and Training Programs (2008). Scaling Up, Saving Lives.

[B5] Murphy C, Harber L, Kiplinger N, Stang A, Winkler J (2007). Learning for Performance: a Guide and Toolkit for Health Worker Training and Education Programs.

[B6] McQuide P, Millonzi K, Farrell C (2007). Strengthening Health Professional Associations Capacity Project Technical Brief No 8.

[B7] Yumkella F (2006). Retention of Health Care Workers in Low-Resource Settings: Challenges and Responses Capacity Project Technical Brief No 1.

[B8] Global HRIS Strengthening. http://www.capacityproject.org/hris/.

[B9] Cooley L, Kohl R (2006). Scaling Up – From Vision to Large-scale Change. A Management Framework for Practitioners.

[B10] Implementing Best Practices Consortium (2007). A Guide for Fostering Change to Scale up Effective Health Services.

